# Literary Note

**Published:** 1904-07

**Authors:** 


### LITERARY NOTE.

Polk's Medical Register and Directory of North America for 1904, has been issued and delivered to the subscribers. By this time it must have been examined or referred to sufficiently to impress those who use it with its great importance as a reference book whenever any person wishes to ascertain the name, residence, professional status, and other important data concerning physicians in the United States and Canada.

It is further of great value in supplying information relating to medical societies, hospitals, medical schools, boards of health, examining boards for license or pensions, medical laws, medical corps of the army, navy, or marine hospital service, medical journals, health resorts, and numerous other important organisations or institutions. Not every fact, of course, can be given regarding persons, or societies, or institutions, nor can it be expected that every item will be absolutely correct where so many people are involved, and such a vast number of societies, hospitals and other bodies and establishments are concerned.

It is, however, surprising that such a near approach to perfection has been made,—a fact only possible by reason of experience, each succeeding edition being better than the former. Moreover, if there is inaccuracy the fault lies with the physicians themselves, all having been addressed with requests to verify the published facts.

Polk's register is indispensable for editors, teachers, public officials, librarians, and all persons who find occasion to ascertain the addresses and essential data relating to physicians and institutions.

### LATE NEWS ITEMS.

Dr. Maitland Ramsay, of Glasgow, visited Dr. Lucien Howe during the latter part of June, on his way to the Saint Louis Exposition. Dr. Ramsay was the guest of the American Medical Association at its last meeting, and will return to Scotland in season for the meeting of the British Medical Association at Oxford.

Dr. Nathan Smith Davis, of Chicago, died at his home June 16, 1904, aged 87 years. He was a native of Greene, N. Y., and was a founder of the American Medical Association which, for a number of years, was practically dominated by his influence and personality. Of late years, however, he had not attended the meetings by reason of increasing feebleness.

